# The SOX11 transcription factor is a critical regulator of basal-like breast cancer growth, invasion, and basal-like gene expression

**DOI:** 10.18632/oncotarget.7437

**Published:** 2016-02-17

**Authors:** Jonathan H. Shepherd, Ivan P. Uray, Abhijit Mazumdar, Anna Tsimelzon, Michelle Savage, Susan G. Hilsenbeck, Powel H. Brown

**Affiliations:** ^1^ Department of Molecular and Cellular Biology, Baylor College of Medicine, Houston, TX 77030, USA; ^2^ The Lester and Sue Smith Breast Center, Baylor College of Medicine, Houston, TX 77030, USA; ^3^ Department of Clinical Cancer Prevention, The University of Texas M.D. Anderson Cancer Center, Houston, TX 77030, USA

**Keywords:** basal-like breast cancer, transcription factor, SOX11, growth, migration

## Abstract

Basal-like breast cancers (BLBCs) are aggressive breast cancers associated with poor survival. Defining the key drivers of BLBC growth will allow identification of molecules for targeted therapy. In this study, we performed a primary screen integrating multiple assays that compare transcription factor expression and activity in BLBC and non-BLBC at the RNA, DNA, and protein levels. This integrated screen identified 33 transcription factors that were elevated in BLBC in multiple assays comparing mRNA expression, DNA cis-element sequences, or protein DNA-binding activity. In a secondary screen to identify transcription factors critical for BLBC cell growth, 8 of the 33 candidate transcription factors (TFs) were found to be necessary for growth in at least two of three BLBC cell lines. Of these 8 transcription factors, SOX11 was the only transcription factor required for BLBC growth, but not for growth of non-BLBC cells. Our studies demonstrate that SOX11 is a critical regulator of multiple BLBC phenotypes, including growth, migration, invasion, and expression of signature BLBC genes. High SOX11 expression was also found to be an independent prognostic indicator of poor survival in women with breast cancer. These results identify SOX11 as a potential target for the treatment of BLBC, the most aggressive form of breast cancer.

## INTRODUCTION

Breast cancer is a heterogeneous disease that can be divided into clinically defined subtypes including estrogen receptor (ER)-positive, HER2-positive, and triple-negative breast cancer (TNBC, which lack ER, progesterone receptor (PR) and HER2), or molecularly defined subtypes including luminal A, luminal B, HER2-enriched and basal-like breast cancer (BLBC). Identifying critical regulators specific to these subtypes has led to the development of targeted therapies; particularly, endocrine therapy for ER-positive breast cancer patients [1], and HER2-targeting therapies for patients with HER2-amplified tumors [2]. As TNBCs lack ER, PR, and HER2, they do not respond to these available targeted therapies. Furthermore, key regulators of TNBC tumor growth have not been defined. Genomic studies of TNBC have revealed that approximately 75% of TNBC can be molecularly defined as BLBC by mRNA expression [3]. Additionally, BLBCs characteristically overexpress proliferation genes and have TP53 gene mutations [3], which contribute to the aggressive growth and poor survival of BLBC [4]. To develop effective targeted therapies for this aggressive type of breast cancer, a better understanding of the key regulators of BLBC is required.

Microarray expression studies are useful in defining distinct breast cancer subtypes. The contrasting mRNA expression in BLBC versus other breast cancer subtypes suggests that transcription factors (TFs) may be critical for the development and progression of these breast cancer subtypes. The important role of transcription factors in promoting breast tumor growth is seen in the luminal subtype which is predominantly defined by the activity of the ER-alpha transcription factor.

In this study we hypothesized that specific transcription factors are critical for BLBC growth and maintenance of BLBC phenotypes. We utilized an integrated genomic screen to identify transcription factors overexpressed or active in BLBC compared to non-BLBC. For this study, we used three independent approaches in our primary screen to identify critical transcription factors by determining the: (1) transcription factors differentially expressed at the mRNA level in TNBC compared with non-TNBC tumors; (2) transcription factor DNA motifs overrepresented among the promoters of selected BLBC genes; and (3) transcription factor motifs which are more highly bound by nuclear proteins present in BLBC versus non-BLBC cells. We then integrated the results of these three independent approaches and identified 33 transcription factors that are highly active in BLBCs. We then performed a secondary screen to discover whether the individual transcription factors identified in our primary screen are critical for growth of BLBC cells, and found that inhibition of 8 of the 33 candidates resulted in reduced growth of at least two of three BLBC cell lines tested. Of the 8 transcription factors critical for BLBC growth, inhibition of SOX11 had the most BLBC-specific effect, suppressing growth in all three BLBC cell lines, while minimally affecting growth of three non-BLBC cell lines. Further evaluation of SOX11 demonstrated SOX11 is critical for multiple BLBC phenotypes including invasion, migration, and expression of key BLBC genes. We also found that high SOX11 expression is an independent prognostic marker of poor survival in women with breast cancer. Taken together, these results identify SOX11 as a critical regulator of gene expression in BLBC and a promising target for the treatment of these aggressive breast cancers.

## RESULTS

### RNA-, DNA-, and protein-based assays identify transcription factors highly expressed or active in basal-like breast cancer

**Figure 1:**
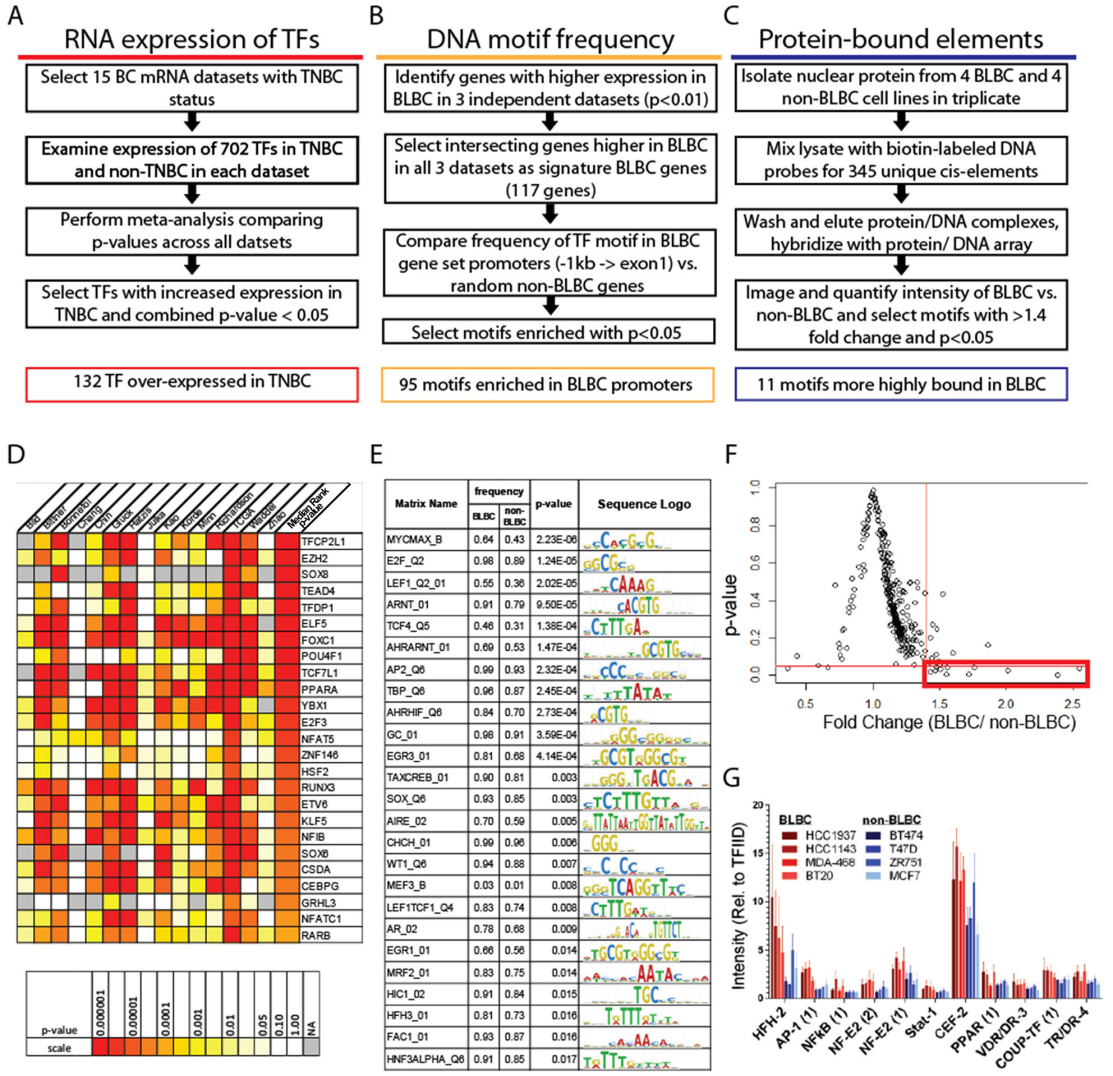
Independent assays of RNA, DNA and protein identify transcription factors increased in BLBC Screening methods used, and number of significant hits identified in **A.** RNA expression screen, **B.** DNA motif screen, and **C.** protein DNA-binding screen. **D.** Heatmap of *p-value*s for 25 most significantly overexpressed transcription factors in TNBC as compared to non-TNBC across 15 datasets, ordered by median rank *p-value*. **E.** Top 25 DNA motifs overrepresented in promoters of BLBC genes. **F.** Results of protein-DNA array by fold change and *p-value*, with the red box indicating motifs with a fold change >1.4 and *p-value* <0.05 in BLBC compared to non-BLBC cells, with individual plots from those candidates in **G.**

To identify transcription factors highly active in BLBCs, we integrated *in silico* and *in vitro* analyses of mRNA expression, DNA sequence, and protein activity (Figure 1). We used mRNA expression to distinguish the set of transcription factors that are more highly expressed in TNBC versus non-TNBC tumors,. We then used DNA sequences from promoters of genes highly expressed in BLBC to identify transcription factor motifs that are overrepresented in BLBC gene promoters. Finally, we tested the DNA-binding activity of nuclear proteins from BLBC and non-BLBC cell lines to identify transcription factor motifs that are more highly bound by proteins from BLBC cells as compared to proteins from non-BLBC cells.

For our RNA-based screen (Figure 1A), we focused on a set of 702 genes whose proteins have been shown to have sequence-specific DNA-binding activity in mammalian cells [5], and examined mRNA expression in TNBC and non-TNBC breast cancer across 15 human breast tumor datasets. As described in Materials and Methods, we used Oncomine™ (oncomine.com, Compendia Bioscience, Ann Arbor, MI) to perform a median-rank-based meta-analysis of differential expression in TNBC vs. non-TNBC. The *p-value*s of each differentially expressed gene in each of the 15 datasets are shown using a colorimetric scale in Figure 1D. The genes were ranked using the *p-value* from the dataset that had the median gene rank across all datasets. The *p-value* of the median ranked gene is shown colorimetrically in the right column labeled “Median Rank *p-value*”. Of the 702 transcription factors queried, we identified 132 transcription factors that are significantly overexpressed (median rank *p-value* <0.05) in TNBC compared to non-TNBC tumors. The top 25 genes are shown in Figure 1D; the complete set of significant genes is listed in Supplementary Table S1.

We next used the frequency of transcription factor DNA binding motifs present in promoters of genes highly expressed in BLBC tumors to identify the transcription factors that regulate the expression of these BLBC genes (Figure 1B). First, we defined a 117-gene BLBC gene set by identifying genes which were more highly expressed in BLBC compared to non-BLBC (with a *p-value* <0.01) in three previously published, independent breast tumor microarrays [6–8] (Supplementary Table S2). Then, using the program CORE_TF [9], we compared the frequencies of individual transcription factor binding motifs within the promoter (defined as 1kb through exon 1) of each gene in the 117-gene BLBC set, as well as 1500 randomly-selected genes that were not significantly overexpressed in BLBC. We identified 95 unique position weight matrices significantly over-represented in promoters of the BLBC gene set with a *p-value* <0.05 using Fisher's Exact Test (the 25 most significantly different motifs are shown in Figure 1E; the complete results for significantly over-represented motifs is shown in Supplementary Table S3). We then used TRANSFAC annotations and published literature to identify 109 unique transcription factor genes that recognize the motifs overrepresented in BLBC gene promoters (Supplementary Table S4).

We conducted a third assay to identify transcription factor proteins that are more active in BLBC versus non-BLBC cell lines by comparing the ability of nuclear proteins from BLBC and non-BLBC cell lines to bind specific DNA response elements. We extracted nuclear protein from 4 BLBC and 4 non-BLBC cell lines and investigated *in vitro* binding of these proteins to specific DNA oligonucleotide motifs to measure DNA-binding activity using transcription factor protein DNA-binding arrays as described in Materials and Methods (and outlined in Figure 1C). Comparing the relative binding of each motif by BLBC and non-BLBC proteins, we used both fold-change (>1.4) and *p-value* (<0.05) cutoff criteria to select transcription factor motifs that had significantly increased binding by protein from BLBC cells (Figure 1F). In these experiments, 11 transcription factor motifs met these criteria, having higher binding by BLBC cells (Figure 1G, full results in Supplementary Figure S1). Using TRANSFAC and published literature, we identified 25 unique transcription factor genes which bind the identified transcription factor motifs (Supplementary Table S5).

### Integration of the three independent assays identifies 33 candidate basal-like breast cancer transcription factors

**Figure 2:**
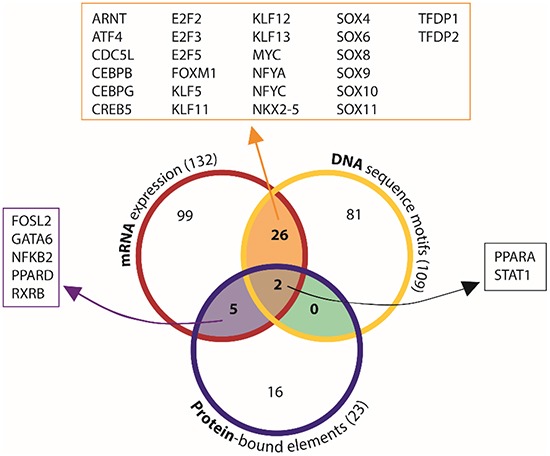
Integration of independent assays identifies 33 candidate BLBC transcription factors Comparison of mRNA expression, DNA sequence motif, and protein-bound element screening results identified transcription factors that were found in multiple assays. A list of transcription factors identified in both the mRNA expression and protein-binding screen (but not DNA-motif) is outlined in purple. A list of transcription factors identified in both mRNA expression and DNA-motif screens (but not protein DNA-binding) is outlined in orange. A list of transcription factors identified in all three screens is outlined in black.

We next integrated the results of the mRNA, DNA, and protein assays from our primary screen to select the set of transcription factors to test in our secondary screen. transcription factors identified in two of the three primary assays were selected for further study (Figure 2). Two transcription factors were identified by all three assays: signal transducer and activator of transcription 1 (STAT1) and peroxisome proliferator-activated receptor alpha (PPARA). An additional 26 transcription factors were identified in both the mRNA expression and DNA-motif frequency screens, while 5 transcription factors were identified in both the mRNA expression and protein-bound motif assays. Thus, a total of 33 transcription factors were identified by at least two of the screening methods. We then investigated each of these 33 transcription factors in our secondary screen to determine which of these transcription factors are required for the growth of BLBC cells.

### Secondary screen to identify transcription factors critical for basal-like breast cancer growth

Our primary integrated genomic screen identified 33 transcription factors highly expressed and active in BLBC cells, but it did not specifically select for growth regulating transcription factors. Therefore, in our secondary screen we identified those transcription factors critical for the growth of BLBC cells. For this screen, we used pools of 3 siRNAs to decrease expression of 29 of the 33 candidate transcription factors in a set of BLBC and non-BLBC cell lines (Table 1) (4 transcription factors were excluded from the secondary screen; we were unable to design effective qRT-PCR assays for 2, and 2 did not exhibit reduced mRNA following knockdown). For the remaining 29 transcription factors, siRNA knockdown was confirmed in MDA-MB-468 cells (data not shown).

**Table 1:** Results of siRNA growth screen of candidate basal-like transcription factors

siRNA	BLBC			Non-BLBC		
	BT20	HCC1143	MDA468	MCF7	T47D	ZR751
SOX11	51 ± 8	61 ± 10	40 ± 7	96 ± 4	72 ± 3	95 ± 10
ATF4	65 ± 19	49 ± 17	47 ± 2	70 ± 7	38 ± 1	78 ± 9
FOXM1	34 ± 5	70 ± 17	52 ± 6	77 ± 11	66 ± 8	105 ± 21
FOSL2	52 ± 11	53 ± 12	71 ± 27	93 ± 21	60 ± 35	84 ± 12
TFDP1	56 ± 11	34 ± 14	103 ± 16	45 ± 8	119 ± 89	123 ± 14
MYC	40 ± 5	34 ± 6	71 ± 11	76 ± 15	42 ± 3	59 ± 14
CDC5L	36 ± 5	25 ± 13	25 ± 9	68 ± 2	30 ± 3	34 ± 6
NFKB2	41 ± 7	23 ± 7	4 ± 4	14 ± 1	28 ± 2	91 ± 14
E2F2	44 ± 11	68 ± 12	79 ± 16	55 ± 15	62 ± 46	77 ± 9
KLF5	74 ± 3	71 ± 7	55 ± 5	57 ± 16	79 ± 11	109 ± 8
RXRB	36 ± 5	70 ± 8	93 ± 21	87 ± 33	28 ± 4	68 ± 9
SOX6	55 ± 7	70 ± 10	89 ± 9	100 ± 5	43 ± 1	76 ± 6
E2F5	66 ± 17	107 ± 31	173 ± 9	140 ± 10	49 ± 3	120 ± 10
NFYA	70 ± 6	74 ± 11	65 ± 6	83 ± 29	101 ± 7	62 ± 11
CEBPB	98 ± 18	71 ± 15	56 ± 8	67 ± 5	76 ± 21	38 ± 12
CEBPG	70 ± 4	86 ± 19	55 ± 4	103 ± 5	96 ± 9	82 ± 6
SOX4	76 ± 17	61 ± 8	90 ± 9	80 ± 10	71 ± 11	91 ± 5
KLF11	122 ± 32	60 ± 13	88 ± 14	88 ± 9	77 ± 19	106 ± 5
KLF13	65 ± 7	87 ± 4	83 ± 20	100 ± 7	81 ± 10	90 ± 7
NFYC	73 ± 12	72 ± 7	78 ± 8	105 ± 13	110 ± 40	39 ± 12
SOX9	91 ± 15	94 ± 15	99 ± 7	108 ± 18	66 ± 10	64 ± 7
SOX10	102 ± 24	81 ± 16	132 ± 9	154 ± 16	32 ± 4	116 ± 9
CREB5	84 ± 15	70 ± 26	99 ± 13	111 ± 14	33 ± 1	117 ± 6
PPARD	76 ± 17	67 ± 9	118 ± 13	88 ± 22	56 ± 8	78 ± 13
ARNT	131 ± 36	153 ± 29	138 ± 12	132 ± 9	22 ± 2	105 ± 12
STAT1	108 ± 22	113 ± 7	80 ± 14	83 ± 6	56 ± 8	93 ± 16
PPARA	103 ± 12	131 ± 26	107 ± 5	94 ± 7	97 ± 10	81 ± 7
KLF12	119 ± 13	79 ± 2	91 ± 2	96 ± 10	79 ± 13	107 ± 6
E2F3	76 ± 5	138 ± 17	107 ± 11	83 ± 10	85 ± 11	91 ± 27
Growth relative to siLuc after 6 days	< 33%	33 - 66%	> 66%			

Growth of BLBC and non-BLBC breast cancer cell lines following siRNA inhibition of candidate BLBC transcription factors. Numbers indicate percent growth after 6 days relative to non-targeting siLuc control ± standard deviation.

As expected, many of the candidate transcription factors were not required for growth, or were critical in only a single BLBC cell line, including the two transcription factors identified by all three screens, PPARA and STAT1. These transcription factors may be important regulators of other basal-like phenotypes. For 8 of the candidate transcription factors, siRNA inhibition suppressed growth by at least one-third (33%) compared to non-targeting siLuc control siRNA in at least two of three BLBC cell lines. Of these 8 critical BLBC growth regulators, three (MYC, CDC5L, and NFKB2) were also critical for growth in two of three non-BLBC cell lines tested, demonstrating that these factors are most likely general growth regulators, not uniquely specific to BLBC. An additional four transcription factors (ATF4, FOXM1, FOSL2, and TFDP1) were moderately specific for growth of BLBC, with siRNA causing growth reduction in at least two BLBC cell lines and only one non-BLBC cell lines. MYC [10] and FOXM1 [3, 11] have been previously associated with breast cancer growth. The identification of these known growth regulating transcription factors validates our integrated screening strategy and serves as a positive control for the secondary growth screen. Of the 8 growth regulating factors, SOX11 demonstrated the most BLBC specificity, with siRNA to SOX11 resulting in reduced growth for all three BLBC cell lines, and minimal growth inhibition in three non-BLBC cell lines.

### SOX11 expression is elevated in basal-like and HER2 breast tumors

**Figure 3:**
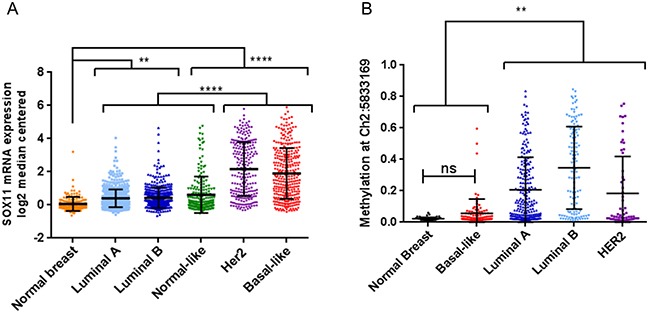
SOX11 expression and DNA methylation varies between normal breast and different breast cancer subtypes **A.** SOX11 mRNA expression in normal breast samples and tumors categorized by PAM50 status, plotted as individual values with mean and standard deviation of the populations. **B.** CpG DNA methylation at a site near the 5′ region of SOX11 in normal breast samples and breast tumors categorized by PAM50 status plotted as individual values with mean and standard deviation of the populations. Statistical significance is indicated by asterisks (****p<0.0001, ***p<0.001, **p<0.01, *p<0.05, ns = not significant).

SOX11 was one of the transcription factors found to have higher expression in TNBC compared to non-TNBC (Supplementary Table S1), but a detailed analysis of SOX11 expression in normal breast and breast cancer subtypes had not been performed. In a large breast cancer dataset, SOX11 mRNA levels are significantly elevated in each of the intrinsic breast cancer subtypes compared to normal breast samples. Additionally, SOX11 levels in HER2 and BLBC subtypes are significantly higher than tumors of the luminal A, luminal B, normal-like, and claudin-low subtypes (Figure 3A and Supplementary Figure S2). While the majority of TNBC breast tumors are classified as BLBC, the heterogeneity of TNBC has led to additional subtypes within TNBC. Using previously published breast cancer expression data [12], we evaluated SOX11 levels in the TNBC subtypes defined by Lehmann et al. [13] and Burstein et al. [12]. SOX11 levels did not significantly differ between the Lehmann defined groups, whereas, within the Burstein defined groups, SOX11 was significantly higher in the basal-like immune-activated (BLIA) and basal-like immune suppressed (BLIS) groups compared to other TNBC subtypes (Supplementary Figure S2).

Several potential mechanisms may be responsible for the elevated SOX11 expression in breast cancer compared to normal breast tissue. One possibility is that SOX11 is methylated in normal adult mammary cells, and becomes hypomethylated in cancer, particularly Her2-positive and basal-like breast cancers. Hypomethylation of oncogenic genes has been associated with many cancer types [14]; and methylation has been shown to be associated with SOX11 expression in hematopoietic and solid tumors [15, 16]. Therefore, we investigated methylation of CpG sites near the SOX11 genomic location. Using methylation and expression data from normal breast samples and breast tumors collected in TCGA, we found that while SOX11 expression is kept low in normal breast samples, CpG sites near SOX11 are not highly methylated (Figure 3 and Supplementary Figure S3). Methylation at the same site in basal-like breast tumors was not significantly altered; however, expression is significantly increased. Interestingly, though most luminal A and luminal B tumors maintain SOX11 expression levels comparable, or slightly higher than normal breast cells, there is a significant increase in methylation levels of CpG sites near SOX11 in these types of tumors (Figure 3 and Supplementary Figure S3).

### SOX11 is critical for the proliferation of estrogen-receptor negative breast cancer cells

**Figure 4:**
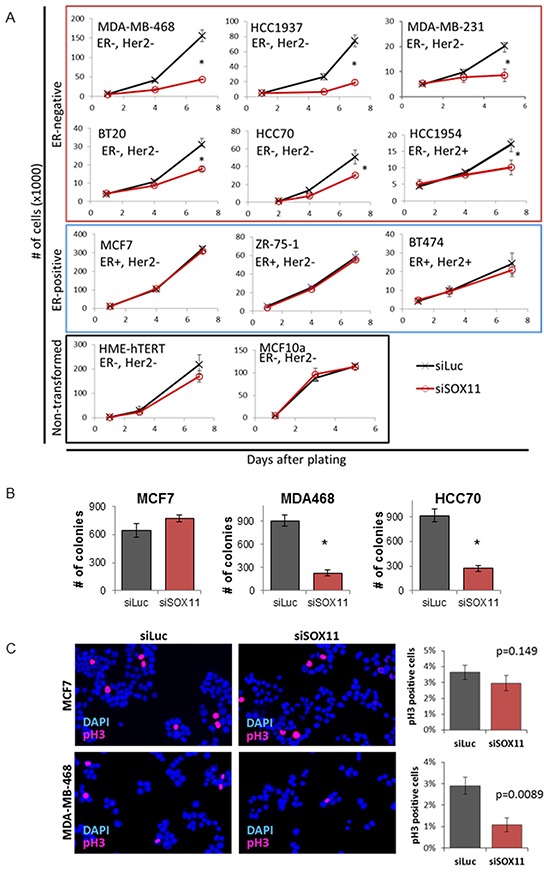
SOX11 is critical for growth and proliferation of BLBC cells **A.** The effect of siRNA depletion of SOX11 on growth of breast cancer and immortalized breast cell lines. Breast cancer cell lines are grouped by ER-status (ER-negtative in top two rows, ER-postive in third row) with immortal, non-transformed breast cells in bottom row. **B.** Colony formation in ER-positive MCF7 and ER-negative MDA-MB-468 and HCC70 cells following siRNA to luciferase (negative control) or SOX11. **C.** Immuno-fluorescent images of MCF7 (top) and MDA-MB-468 (bottom) following non-targeting siLuc or SOX11 targeted siRNA stained for phosphorylated histone H3. Representative images shown with quantification taken from three independent replicates, with summed counts from seven unique fields for each sample. * denotes *p*<0.05.

To investigate the role of SOX11 in regulating the growth of breast cancer cells, we used siRNA to inhibit SOX11 and measure two-dimensional growth *in-vitro* in a panel of ER-negative/HER2-negative (BT20, MDA-MB-468, HCC70, HCC1937, and MDA-MB-231), ER-negative/HER2-positive (SKBr-3, and HCC1954), ER-positive/HER2-positive (BT474), and ER-positive/HER2-negative (MCF7 and ZR751) breast cancer cell lines as well as non-transformed breast cell lines (HME-hTERT and MCF10A). SOX11 knockdown significantly reduced growth of each of the ER-negative breast cancer cell lines tested, including both HER2-negative and HER2-positive, ER-negative cell lines, as well as triple-negative cell lines representative of the claudin-low subtype. (Figure 4A, top rows). Alternatively, SOX11 knockdown had little to no effect on the growth of ER-positive breast cancer cells or non-transformed breast cells (Figure 4A, lower rows), which are ER-negative, but did not have detectable levels of SOX11 by qPCR (data not shown). We also evaluated the effect of SOX11 inhibition on anchorage-independent growth and found that siRNA knockdown of SOX11 did not inhibit colony formation in the ER-positive cell line MCF7 (Figure 4B, left), but did significantly inhibit colony formation in ER-negative cell lines MDA-MB-468 and HCC70 (Figure 4B, center and right). These findings demonstrate that SOX11 is required for growth of ER-negative breast cancer cell lines, but is not required for growth of ER-positive breast cancer cells, or non-transformed breast cell lines.

To evaluate whether loss of SOX11 results in decreased proliferation of BLBC, phosphorylated histone H3 (pH3), a marker of mitotic cells, was measured in MCF7 and MDA-MB-468 cells which had been treated with siRNA targeting SOX11 or non-targeting control siRNA. SOX11 knockdown did not significantly decrease the proportion of pH3-positive cells in MCF7 cells, but did have a significant effect, reducing the percent of cells which stained positive for phosphorylated Histone H3 in MDA-MB-468 cells (Figure 4C), demonstrating that loss of SOX11 in BLBC cells reduces proliferation. Conversely, SOX11 knockdown did not significantly increase apoptosis in BLBC cells as measured by Annexin-V flow cytometry (Supplementary Figure S4), demonstrating that apoptosis did not contribute to the decrease in growth of this cell line.

### SOX11 promotes increased migration of breast cancer cells

While investigating the role of SOX11 depletion in ER-negative breast cancer cells, we noted an altered morphology of MDA-MB-231 cells following treatment with siRNA targeting SOX11. MDA-MB-231 cells typically have a mesenchymal-like appearance with long protrusions; however, following SOX11 depletion, the cells appear more cuboidal (Supplementary Figure S5, upper panel). We therefore investigated whether SOX11 played a role in maintaining the epithelial-to-mesenchymal transition (EMT)-like characteristics associated with BLBC [17], including increased motility and invasiveness as well as expression of transcription factors previously shown to promote EMT-like characteristics.

**Figure 5:**
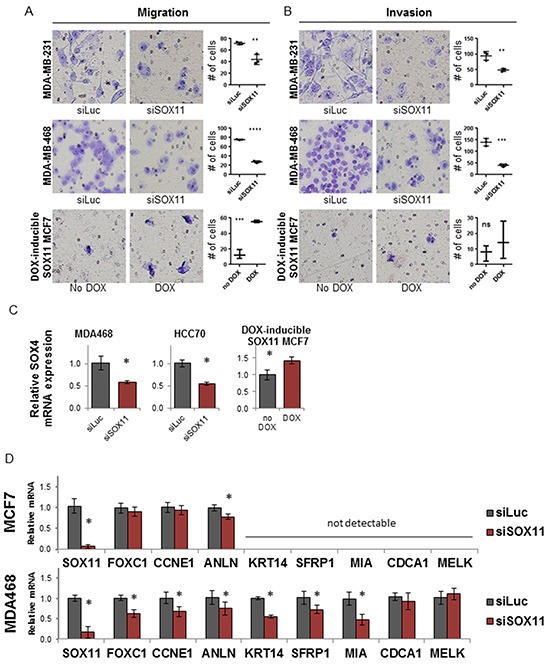
SOX11 is a critical regulator of migration, invasion, and BLBC gene expression Representative pictures and quantification of the effect of SOX11 siRNA on migration **A.** and invasion **B.** for MDA-MB-231 (top row), MDA-MB-468 (middle row) and MCF7 cells with DOX inducible SOX11 expression (bottom row). **C.** Relative mRNA expression of SOX4 in ER-negative MDA-MB-468 and HCC70 cells following siRNA knockdown of SOX11 (left and middle) and ER-positive MCF7 cells with DOX-induced overexpression of SOX11. **D.** Relative expression of SOX11 and a sampling of PAM50 genes associated with BLBC following non-targeting siLuc or SOX11 inhibiting siRNA in MCF7 and MDA-MB-468 cell lines. * denotes *p*<0.05.

For these studies, cell migration and invasion of breast cancer cell lines MDA-MB-468 and MDA-MB-231 was measured following siRNA inhibition of SOX11, whereas the ER-positive cell line, MCF7, which typically demonstrates low levels of motility and invasion, was transduced with a viral vector encoding the SOX11 cDNA under the control of a tetracycline-inducible promoter to produce overexpression of SOX11 upon the addition of doxycycline (DOX). The ability of the invasive, ER-negative breast cancer cells lines to migrate towards serum supplemented media either uninhibited (Figure 5A, top two rows) or through a matrigel coated membrane (Figure 5B, top two rows) was reduced upon knockdown of SOX11. A reduction in migration was also seen in other ER-negative cell lines: HCC70, HCC1937, and HCC1954 (Supplementary Figure S5 lower panel). This result indicates that SOX11 is critical for the increased motility associated with aggressive BLBC cells. Overexpression of SOX11 in MCF7 cells significantly increased the migratory ability of the cells (Figure 5A, bottom row), and caused a small but non-statistically-significant increase in the invasiveness of the cells (Figure 5B, bottom row). These results suggest that SOX 11 primarily promotes promotes the migratory ability of breast cancer cells.

Following SOX11 depletion, ER-negative cells exhibited a reduction in the closely related SOX transcription factor, SOX4, (Figure 5C, left and center), whereas overexpression of SOX11 in MCF7 cells resulted in an increase in SOX4 expression (Figure 5C, right). SOX4 has been shown to function cooperatively with SOX11 during neuronal development [18] and is also a regulator of EMT in breast cancer [19]. These findings suggest that SOX11 may cooperate with other SOX factors and regulators of migration and invasion to promote EMT-like characteristics associated with aggressive basal-like breast tumors.

### SOX11 is critical for elevated expression of genes that define the basal-like subtype

Breast tumors can be subtyped into BLBC and the other molecular subtypes using the Prediction Analysis of Microarray 50 (PAM50) test, which analyzes expression of 50 genes to separate breast tumors into specific subtypes [20]. We next investigated whether SOX11 inhibition would alter expression of the genes that define the BLBC subtype, particularly those genes in the PAM50 test which are characteristically high in BLBC tumors. Therefore, the expression of several PAM50 genes (FOXC1, CCNE1, ANLN, KRT14, SFRP1, MIA, CDCA1, MELK) was measured following inhibition of SOX11 (Figure 5D).

In the non-BLBC cell line MCF7, most signature BLBC genes tested were either undetectable by qRT-PCR, or did not change following SOX11 depletion. The exception to this was anillin, actin binding protein (ANLN), which was reduced in both the MCF7 cell line and the BLBC cell line MDA-MB-468 following SOX11 depletion. In MDA-MB-468 cells, the majority of signature BLBC genes were reduced following inhibition of SOX11. This set of genes included forkhead box C1 (FOXC1), keratin 14 (KRT14), cyclin E1 (CCNE1), melanoma inhibitory activity (MIA) and secreted frizzled-related protein 1 (SFRP1), while expression of CDCA1 and MELK, (neither of which were detectable in MCF7), did not change following SOX11 depletion. These results show that SOX11 regulates the expression of many genes that define “basal-ness” in BLBC.

### High SOX11 expression is associated with poor survival

**Figure 6:**
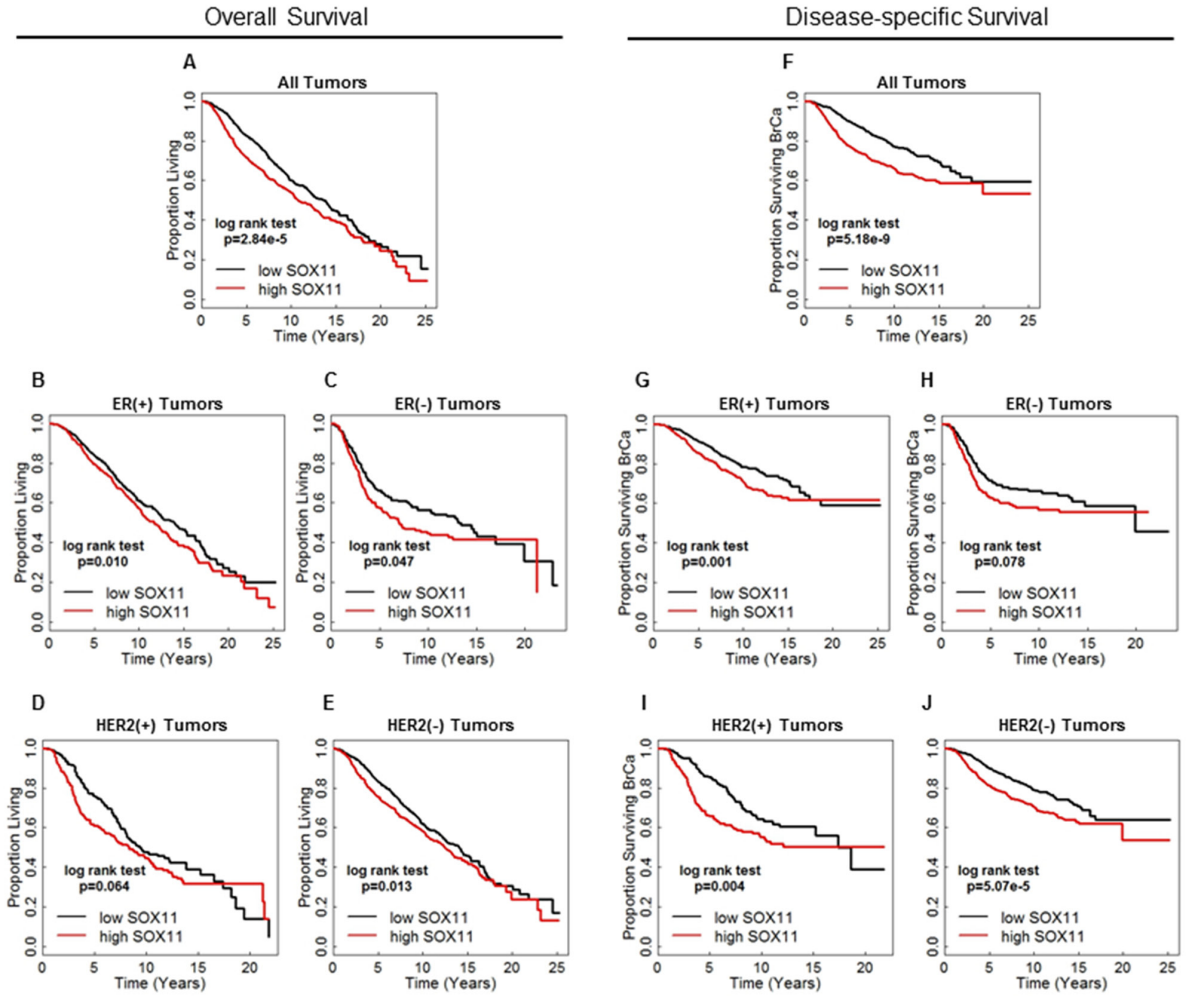
High SOX11 associates with poor prognosis Survival data from the Curtis dataset [50] investigating overall survival in all patients **A.** or subsets of patients based on ER-status **B–C.** or HER2 status **D–E.** with patients dichotomized by the median SOX11 expression level within each analysis. Equivalent studies of breast cancer-specific survival for all patients **F.** or subsets based on ER-status **G–H.** and HER2 status **I–J.** were also performed.

We next investigated whether SOX11 is a prognostic marker in women with breast cancer by performing survival analyses using multiple available datasets as described in Materials and Methods. This analysis showed that high SOX11 expression is significantly associated with poor disease-specific, overall, recurrence-free, and metastasis-free survival in multiple breast cancer datasets (Figure 6A and Supplementary Figure S6). As ER status is known to be strongly correlated with breast cancer survival, we used the Curtis dataset, which had the most patients with follow-up survival data, to also examine the correlation of SOX11 with survival among patients after stratifying by ER or HER2 status. In analyses of overall survival (Supplementary Figure S6), high SOX11 was associated with worse prognosis for patients with either ER-negative or ER-positive tumors and was also correlated with poor survival regardless of HER2 status. Using disease-specific survival outcome (Figure 6F–6J), high SOX11 was suggestive of worse disease-free survival in ER-negative patients (Figure 6H), but this result only trended toward significance (*p*=0.0775), and was most likely due to the small sample size of patients with ER-negative breast cancer available for the disease specific survival analysis. While SOX11 levels are comparatively low in ER-positive tumors (Figure 2A), ER-positive tumors with higher than median SOX11 expression have a significantly worse prognosis. High SOX11 expression also correlated with poor disease-specific survival regardless of HER2 status. These results suggest that though SOX11 is typically more highly expressed in basal-like and HER2-positive breast cancer, it likely functions in other breast cancer subtypes to promote the growth of aggressive, poor-prognosis tumors. To formally determine the prognostic value of SOX11 expression in breast cancer survival, a multivariate Cox proportional hazards analysis including SOX11 expression, tumor size, grade, node status, and PAM50 subtype was utilized and demonstrated that SOX11 expression is an independent prognostic indicator for increased risk of breast cancer related death (Table 2).

**Table 2:** High SOX11 is an independent prognostic factor associated with increased risk of breast cancer caused death

	Disease-Specific Survival - Curtis Dataset		
	n	HR (conf. int)	*p-value*
**SOX11 Low**	996	Reference	
**SOX11 High**	996	**1.42** (1.17-1.73)	**0.00052**
**Tumor Size**		**1.01** (1.01-1.02)	**<0.00001**
**Grade 1**	170	Reference	
**Grade 2**	775	1.54 (0.94-2.54)	0.08609
**Grade 3**	957	**1.69** (1.03-2.79)	**0.03852**
**Grade NA**	90		
**Lymph neg**	1042	Reference	
**Lymph pos**	950	**2.22** (1.83-2.69)	**<0.00001**
**Luminal A**	721	Reference	
**Luminal B**	492	**2.04** (1.57-2.64)	**<0.00001**
**Normal-like**	202	**1.71** (1.18-2.46)	**0.00456**
**HER2**	240	**2.21** (1.63-3.00)	**<0.00001**
**Basal-like**	331	**1.84** (1.35-2.51)	**0.00011**
**Not classified**	6		

Multivariate Cox proportional hazards model of the risk associated with high SOX11 expression on disease- specific survival in Curtis Dataset [50], including tumor size, grade, lymph node status and breast cancer subtype.

## DISCUSSION

In this study we used a novel screening approach integrating RNA, DNA and protein data to identify transcription factors which are highly expressed and active in BLBC. For this study, we focused on transcription factors which were critical for growth in BLBC cell lines and identified 8 transcription factors that were critical for the growth of BLBC. One of these transcription factors, SOX11, is a novel regulator of BLBC growth which regulates multiple BLBC phenotypes, including migration, invasion, and expression of genes highly expressed in BLBC tumors. Furthermore, we found that high SOX11 expression is associated with poor breast cancer patient survival. Therefore, our studies demonstrate that SOX11 may be a master regulator of the BLBC subtype and a novel target for the treatment of this aggressive disease.

Each of the assays in the primary screen identified transcription factors potentially involved in regulating critical processes in BLBC cells. Among these are several transcription factors previously described as highly expressed in BLBC or TNBC, including FOXC1 [21], EZH2 [22], and ELF5 [23], (identified in our mRNA expression analysis); MYC [3, 10], E2F factors [24], and HIF1/ARNT [3, 25] (identified in our DNA promoter sequence analysis); and AP-1 [26] and NF-kB [27] (identified in our protein DNA-binding assay). The identification of these transcription factors known to be highly expressed in BLBC serves to demonstrate the validity of our screen.

Focusing this study on transcription factors critical for growth excluded the two transcription factors which were discovered by all three screens, PPARα and STAT1. While our study indicates that PPARα and STAT1 are not essential for BLBC growth, these factors may be critical in the development and maintenance of other BLBC phenotypes. PPAR transcription factors are known regulators of lipid and fatty acid metabolism (reviewed in [28]), and understanding the role of PPARα in BLBC may require investigation of lipid metabolism within mammary epithelial cells as well as in conjunction with adjacent adipocytes. The role of STAT1 in breast cancer is not fully understood, with studies suggesting both tumor suppressing [29], and tumor promoting activities [30]. Indeed, loss of STAT1 in mice results in spontaneous development of ER-positive breast tumors [31], whereas expression of STAT1 was found to be increased in node-positive TNBC compared to node-negative TNBC. These results suggest that STAT1 may act as a tumor suppressor in ER-positive breast cancer cells, but may promote invasiveness of TNBCs.

We identified 8 transcription factors for which siRNA inhibition reduced growth in multiple BLBC cell lines in our secondary screen. Of these, only SOX11 was required for growth of all 3 BLBC cell lines, but not for the growth of the non-BLBC cell lines. SOX11 is a member of the SOX (SRY-related HMG-box) family of transcription factors. Other members of this family, including SOX9 [32], SOX10 [33], and SOX4 [19], have been shown to be important regulators of mammary stem cells and EMT; whereas, fewer studies have evaluated the role of SOX11 in normal or malignant breast cells. SOX11 has been previously shown to be highly expressed in embryonic mammary bud epithelial cells [34], and SOX11 was elevated in BRCA−/− mammary epithelium compared to mammary epithelium in normal postnatal mice [34]. The role of SOX11 in other cancers has been particularly dependent on cancer type, with reports suggesting both oncogenic and tumor suppressor roles. In mantle cell lymphoma (MCL) SOX11 is highly expressed and is a diagnostic biomarker that distinguishes MCL from other mature B-cell lymphomas [35]. Further study of SOX11 in MCL cells has demonstrated that high SOX11 promotes the growth of lymphoma cells and prevents differentiation [36]. However, in other cancer types, including other hematological cancers, and epithelial ovarian cancer, SOX11 is frequently methylated and high SOX11 expression is associated with improved survival [16, 37–39]. Our results demonstrate that SOX11 is differentially expressed in breast cancer subtypes and is required for the growth of transformed ER-negative breast cancer cells, but not for the growth of ER-positive cancer cells or non-transformed breast cells. These results indicate that the role of SOX11 needs to be considered within specific cell and cancer types.

Several studies have shown that SOX11 can regulate differentiation. In MCL and in osteoblasts, SOX11 promotes proliferation of progenitor cells and blocks terminal differentiation [36, 40]. Our findings suggest that SOX11 may serve a similar role in BLBC, promoting growth, preserving migratory and invasive characteristics associated with less differentiated breast cells, and preventing differentiation to luminal cells. Additionally, SOX11 and other members of the SOXC group, have been shown to be required for survival of progenitor cells during organogenesis [41]. Therefore, the role of SOX11 in breast development and differentiation is an important subject for future investigation.

Our results demonstrating that SOX11 inhibition affected multiple BLBC phenotypes suggest that SOX11 acts as a key signaling molecule in these breast cancers. This theory is supported by our finding that several PAM50 markers of BLBC, including the FOXC1 transcription factor, are reduced following SOX11 inhibition. High FOXC1 mRNA expression is frequently seen in BLBCs [21]. FOXC1 has been shown to be enriched in mammary progenitor cells [42], and its reduction in response to SOX11 inhibition in BLBC may be critical for the shift towards the BLBC phenotype.

Our results showed that high SOX11 expression is correlated with worse clinical outcome in breast cancer patients. However, other studies in ovarian cancer [37] and in gastric cancer [43] have found that elevated SOX11 is associated with improved outcome, and that SOX11 can act as a tumor suppressor. These findings demonstrate that the roles of SOX11 are likely to be dependent on the context of the tumor and cell type. In breast cancers, one group has recently shown that high grade breast tumors had lower levels of nuclear SOX11 protein, and that nuclear SOX11 was associated with improved clinical outcome [44]. Our results reported here combined with these previous studies support further evaluation of the role and clinical significance of SOX11.

For this study we used a novel, comprehensive, and integrated approach utilizing RNA-, DNA- and protein-based assays to identify a set transcription factors that are differentially activated in BLBC and may be critical for BLBC development and progression. The discovery that SOX11 is critical for the growth, migration, and invasion of BLBC cells identifies a novel molecule that can be targeted for the treatment of BLBC. Further study of SOX11 and the other transcription factors identified here will be essential to understanding their role controlling BLBC growth and tumorigenicity. Such studies will provide additional therapeutic targets to effectively treat this aggressive form of breast cancer.

## MATERIALS AND METHODS

### Cell lines

Breast cancer cell lines were purchased from American Type Culture Collection (ATCC, Manassas, VA) and their identities were confirmed by short tandem repeat (STR) DNA fingerprinting using the AmpFℓSTR Identifiler kit (cat# 4322288, Life Technologies, Foster City, CA). as described previously [45]. Cells were maintained according to ATCC recommendations.

### RNA expression of transcription factors in TNBC and non-TNBC

Expression of 702 genes previously identified as DNA-binding transcriptional regulators [5] was compared using Oncomine™ (oncomine.com, Compendia Bioscience, Ann Arbor, MI) to perform a meta-analysis of differential expression between TNBC and non-TNBC across 15 breast tumor datasets which contain TNBC status annotation (listed in Supplementary Table S1). Differential gene expression analysis was performed for each dataset, with all genes ranked by significance (*p-value*) for higher expression in TNBC vs. non-TNBC tumors. The *p*-values for each dataset are shown using a colorimetric scale in Figure 1D. The *p*-value of the dataset showing the median gene rank across all datasets is shown on the right in the column labeled “Median Rank *p-value*.” The median gene rank and associated *p-value* across the set of 15 datasets for each investigated transcription factor was then used to select candidate transcription factors which had increased expression in TNBC with a cutoff of *p*<0.05.

### DNA Cis-element promoter analysis

mRNA expression and tumor subtype classifications from three previously published breast tumor microarray studies [6–8] were used to select genes consistently more highly expressed in BLBC compared to non-BLBC. From each dataset, we selected the genes with higher expression and a univariate *p-value* <0.01 in BLBC tumors compared to non-BLBC tumors. The intersection of the three resulting sets gave a 117-gene BLBC gene set. A set of 1500 control genes were randomly selected from the set of genes not significantly more highly expressed in BLBC tumors. The online tool, CORE_TF [9], was then used for cis-element recognition using binding matrices from TransFac 11.2 (Biobase, Beverly, MA) [46] in the region from −1 kb through exon 1 of each gene. The frequency of motif occurrence among the 117 BLBC gene promoters was compared to that of the same region in control gene promoters to identify response elements significantly enriched in genes highly expressed in BLBC. An exact binomial test with a cutoff of *p*<0.05 was used for significance.

### Transcription factor protein DNA-binding assay

Nuclear proteins were collected from breast cancer cell lines using the NE-PER kit (Thermo Scientific, Rockford, IL). The protein DNA-binding assay was performed using the Combo Protein/DNA Array (Affymetrix cat# MA1215, Santa Clara, CA) according to manufacturer instructions. Briefly, 10ug of nuclear protein was combined with biotin-labeled DNA probe mix representing 345 consensus binding motifs. After incubation, the protein/probe mixture was put through spin columns to remove unbound probes. The protein/probe mixture was heat denatured, and the previously bound probes were hybridized to membranes containing corresponding consensus transcription factor binding motifs. Detection was performed with streptavidin-HRP using BioRad ChemiDoc. TIF images were quantified using ImageJ Dot Blot Analyzer (available in IJ macros toolsets repertory at http://rsb.info.nih.gov/ij/macros/toolsets/DotBlotAnalyzer.txt), with the intensity of each spot calculated relative to TFIID. The experiment was performed in triplicate for each cell line. The BLBC average (relative intensity of each spot averaged across each of the triplicate values for HCC1143, HCC1937, BT20, and MDA468) and the luminal average (relative intensity averaged across each replicate for T47D, MCF7, BT474, and ZR751) were calculated and compared using the Student's t-test. Spots which had a fold change greater than 1.4 and with a *p-value* less than 0.05 were included for follow up.

### Integration of DNA promoter, RNA expression and protein DNA-binding assays

For the promoter analysis and the protein DNA-binding analyses which result in transcription factor motifs, we used TRANSFAC [46] annotation and published literature to identify transcription factors which recognize the identified motifs and specific genes which compose the resulting transcription factors. The set of genes resulting from each assay were compared and we selected the set of 33 genes which were identified in at least 2 of the 3 assays.

### siRNA, quantitative real-time polymerase chain reaction (qPCR) and growth, migration and invasion assays

siRNA, qPCR and growth assays were performed as previously described [47]. qPCR sequences are listed in Supplementary Table S6. Migration and invasion assays were performed as previously reported [48], allowing 18 hours for migration, after which passed cells were stained (Hema 3 System, Fischer Scientific #22-122911) and five 20x fields counted.

### Phospho-histone H3 immunofluorescence

The phospho-histone H3 immunofluorescence assay was performed as previously described [49] with primary anti-phoshpo-histone H3 (Cell Signaling #3377) and secondary Alexa Fluor 594 conjugated anti-rabbit (Life Technologies #A-11037) antibodies. Nuclei were stained with 4′,6-diamidino-2-phenylindole (DAPI). Images were acquired using a Nikon Eclipse Ti microscope with NIS Elements imaging software (Nikon, Melville, NY).

### Analysis of survival in breast cancer datasets

mRNA expression and survival data from Curtis *et al.* [50], Desmedt *et al.* [51], Esserman *et al.* [52], and Hatzis *et al.* [53] were used to evaluate the prognostic importance of SOX11. Data obtained from the Oncomine database were analyzed using R statistical software to generate Kaplan–Meier survival curves, determine statistical significance using the log rank (Mantel–Cox) method, and conduct Cox proportional hazards models analyses. Expression data was dichotomized at the mean expression level.

## SUPPLEMENTAL MATERIAL FIGURES AND TABLES














