# Supplementary material for: The SOX11 transcription factor is a critical regulator of basal-like breast cancer growth, invasion, and basal-like gene expression
Source: Oncotarget. 2016 Feb 17;7(11):13106–21. doi: 10.18632/oncotarget.7437 (PMC4914345; doi:10.18632/oncotarget.7437)
Supplement: Supplementary file 1 [file oncotarget-07-13106-s001.pdf]

SUPPLEMENTARY FIGURES AND TABLES

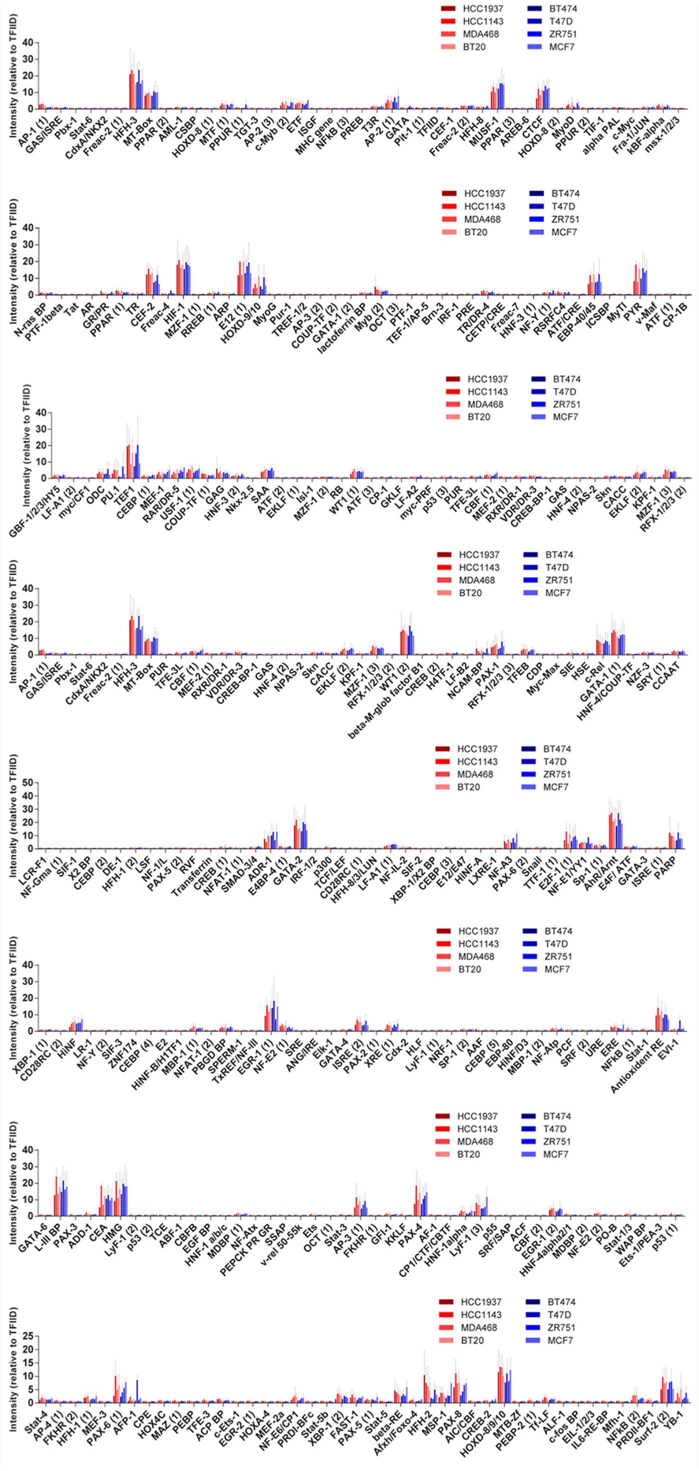

Supplementary Figure S1: Breast cancer cell line nuclear protein-bound oligonucleotide sequences. Average intensities relative to TFIID from 3 independent experiments measuring 345 biotin-labeled oligonucleotides from Affymetrix Combo Protein/DNA Array (Affymetrix, Santa Clara, CA, Cat# MA1215) following incubation with nuclear lysates from either basal-like breast cancer cell lines (depicted in shades of red) or non-basal-like cell lines (depicted in shades of blue).

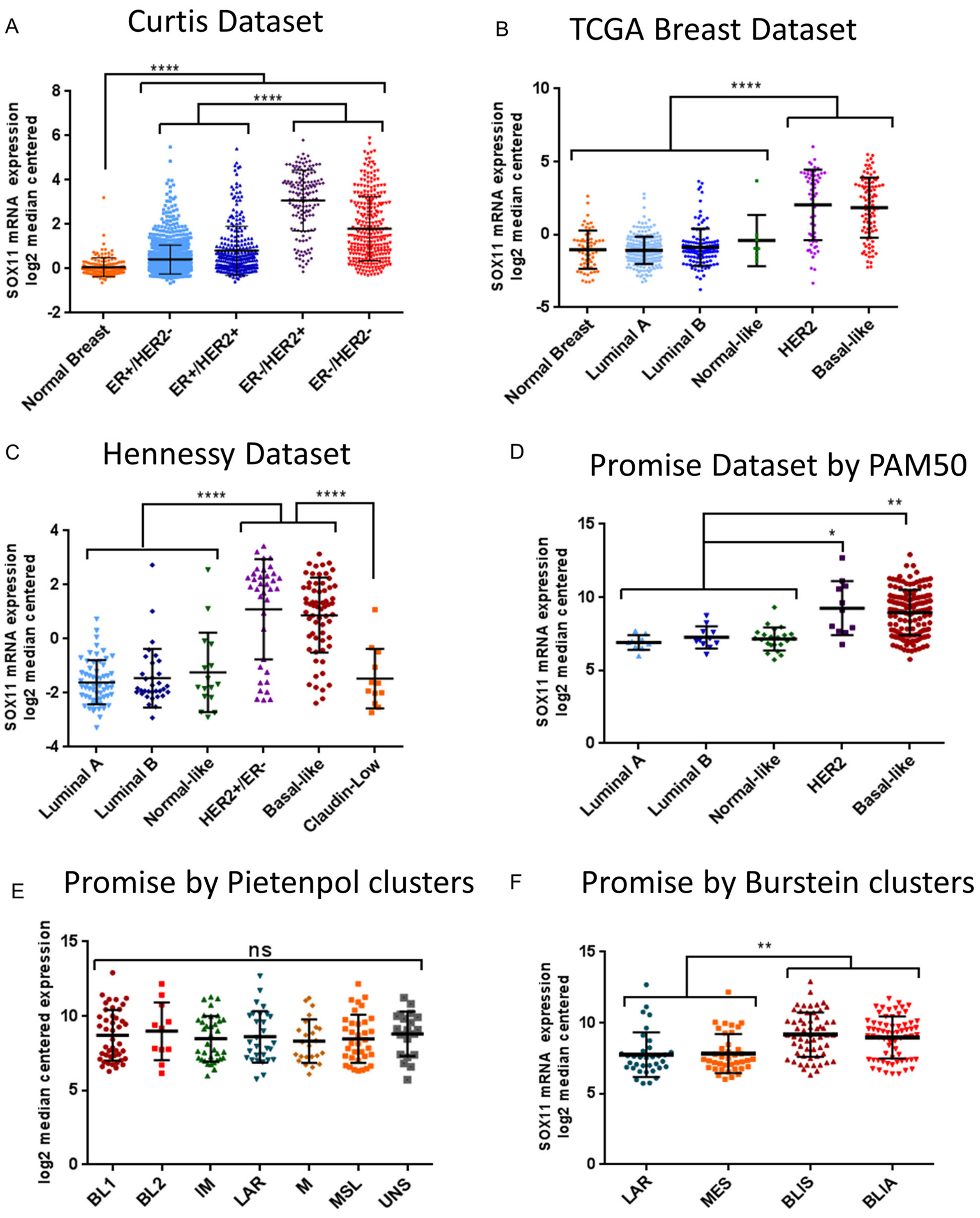

Supplementary Figure S2: SOX11 mRNA expression in multiple breast cancer datasets subsetted by different biomarkers and molecular subtypes. mRNA expression of SOX11 in **A.** the Curtis dataset comparing normal breast samples and breast tumors grouped by ER and HER2 status; **B.** the TCGA breast dataset comparing normal breast and breast tumors divided by PAM50 subtype; **C.** the Hennessy dataset comparing breast tumors by PAM50 Status with the addition of the Claudin-low subtype. The Promise dataset in **D–F.** are from Burstein, *et al* "Comprehensive genomic analysis identifies novel subtypes and targets of triple-negative breast cancer" Clin Cancer Res. 2015 Apr 1;21(7):1688-98. (PMID: 25208879) and compare triple negative breast tumors based on PAM50 subtypes (D), subtpes defined by Lehmann and Pietenpol (E), or those defined by Bustein (F). Statistical significance is indicated by asterisks (****$p < 0.0001$, ***$p < 0.001$, **$p<0.01$, *$p<0.05$, ns = not significant).

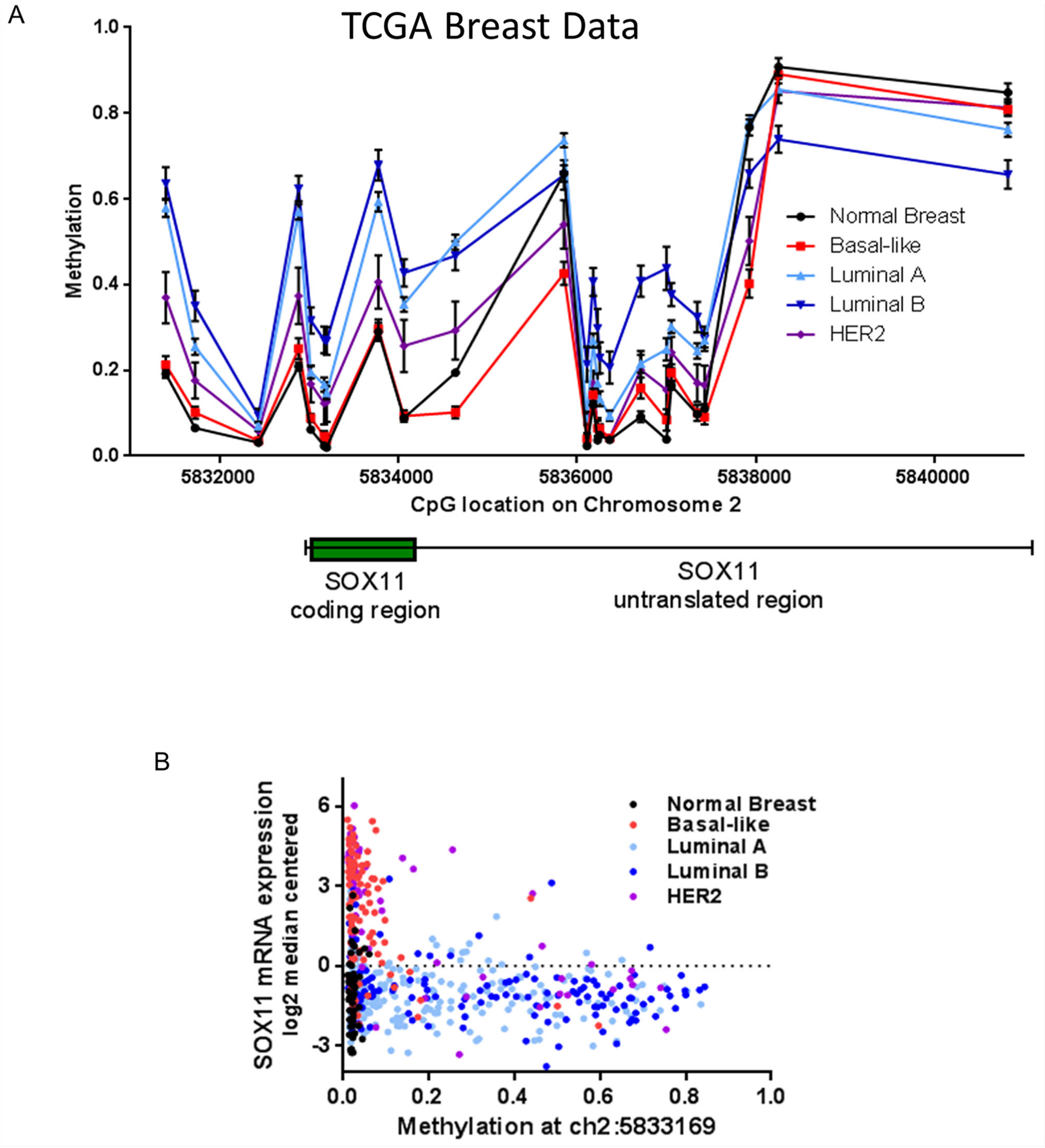

Supplementary Figure S3: SOX11 expression and DNA methylation varies between normal breast and different breast cancer subtypes. A. Methylation for normal breast and breast cancer subtypes at DNA CpG sites near the SOX11 genomic locus, data from TCGA breast dataset. Error bars represent standard deviation. **B.** SOX11 relative expression and CpG DNA methylation at a site near the 5′ region of SOX11, spots are from individual samples which are colored to reflect normal breast samples or breast tumors categorized by PAM50 status.

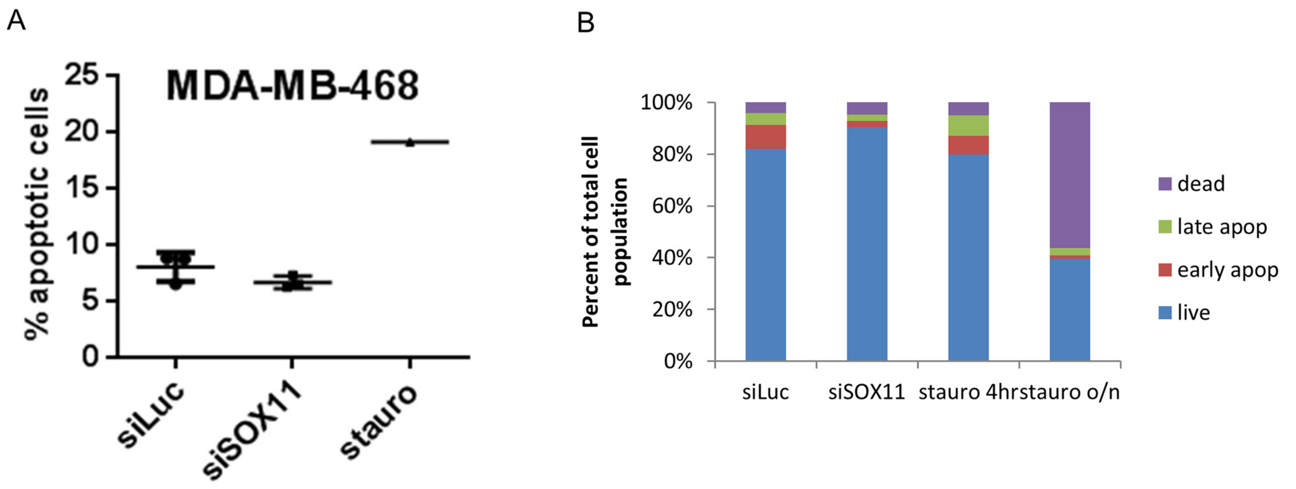

Supplementary Figure S4: Apoptosis quantifiaction following SOX11 depletion in MDA-MB-468 cells. Total proportion of apoptic cells **A.** or the proportions of early and late apoptotic cells **B.** as measured by Annexin V and propidium iodide staining, following control siLuciferase (siLuc) or SOX11 targeting (siSOX11) siRNA. Staurosporin (stauro) was used as a positive control to induce apoptosis.

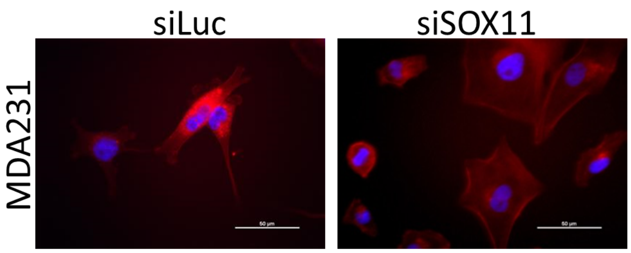

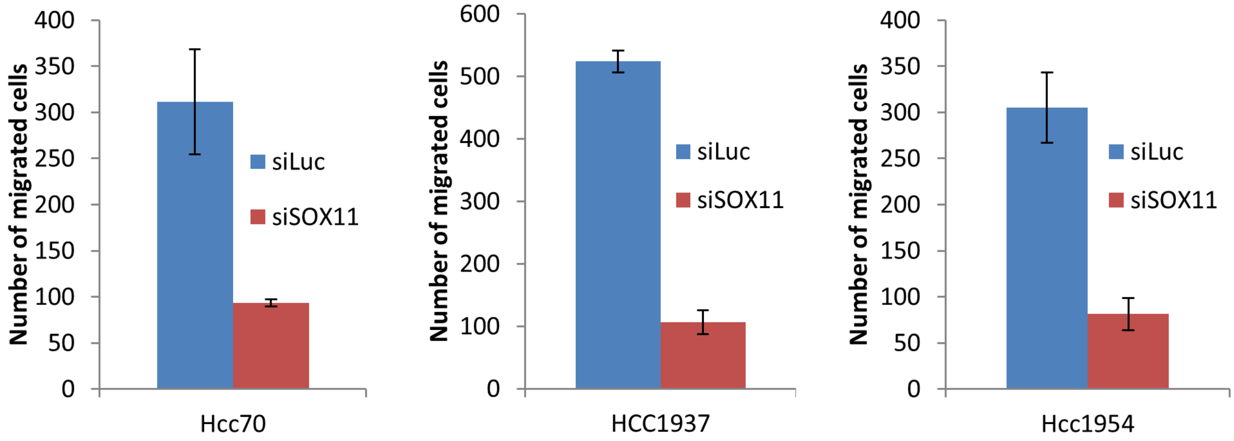

Supplementary Figure S5: SOX11 depletion affects cell morphology and migration. A. MDA-MB-231 cells transfected with either control (siLuc) or SOX11 targeting (siSOX11) siRNA and stained with phalloidin and 4',6-diamidino-2-phenylindole (DAPI). Number of cells migrating through 8um pore transmembrane in 18 hours following transfection with control (siLuc) or SOX11 targeting (siSOX11) siRNA for HCC70 **B.** HCC1937 **C.** or HCC1954 **D.** cells.

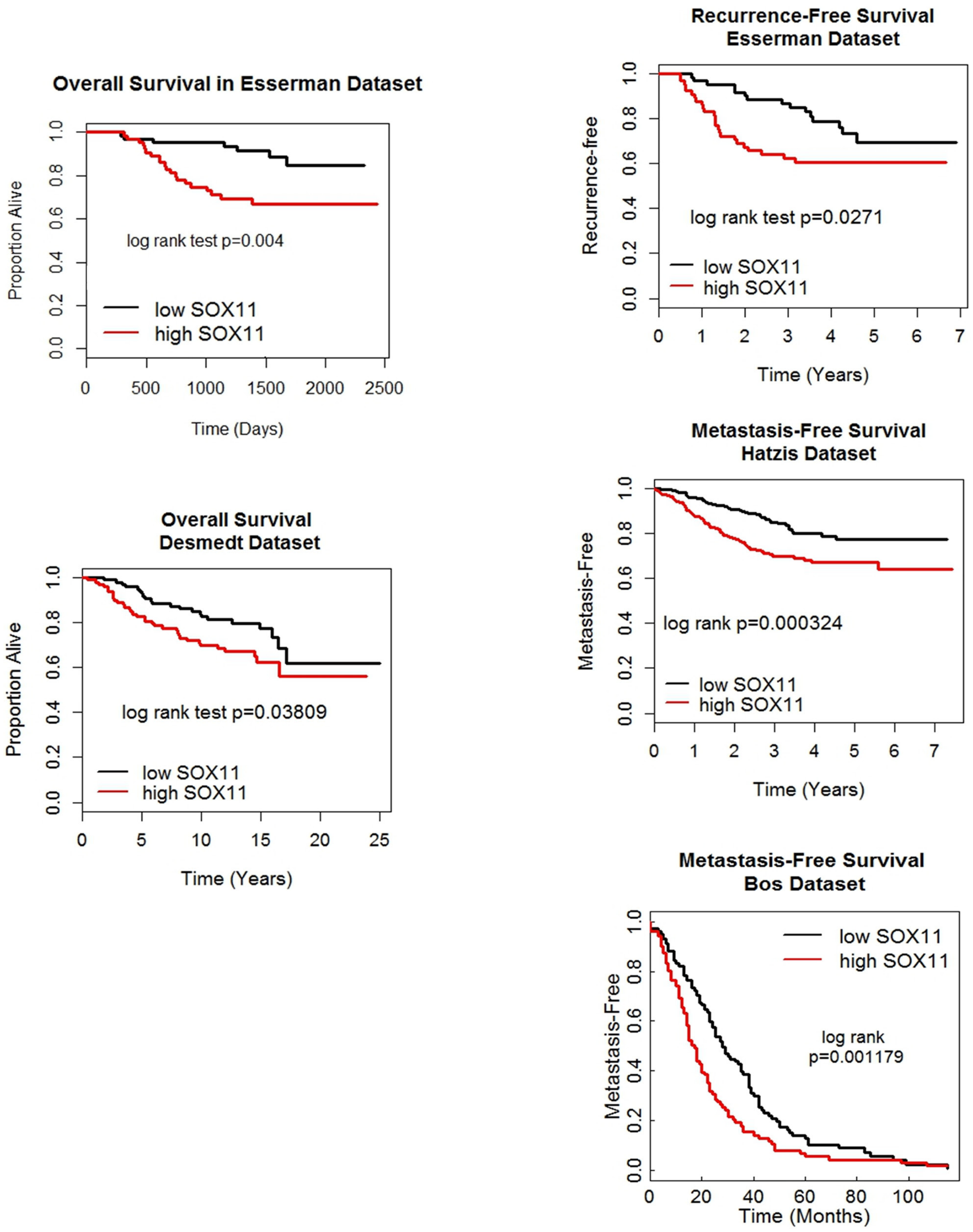

Supplementary Figure S6: High SOX11 expression correlates with poor prognosis in multiple datasets. Higher than mean SOX11 expression significantly associated with shorter overall-survival in the Esserman **A.** and Desmedt **B.** datasets, with shorter recurrence-free survival in the Esserman dataset **C.** and shorter metastasis-free survival in the Hatzis **D.** and Bos **E.** datasets. *p*-valuesshow significance of log rank test.

Supplementary Table S1: Analysis Comparison: Oncomine 15 datasets with TNBC status - expression in TNBC vs non-TNBC.

(See Supplementary File 1)

Supplementary Table S2: 117 Gene basal-like signature. Set of genes identified to be higher in BLBC vs. non-BLBC tumors with $p<0.01$ in all three datasets.

(See Supplementary File 2)

Supplementary Table S3: Motif Frequency analysis - Full results.

(See Supplementary File 3)

Supplementary Table S4: Transcription factor genes with increased motif frequency in promoters of basal-like genes.

(See Supplementary File 4)

Supplementary Table S5: Transcription factor genes with oligo motif more highly bound by nuclear protein from basal-like cell lines.

(See Supplementary File 5)

Supplementary Table S6: qPCR primer and probe sets.

(See Supplementary File 6)
